# Allelopathic Potential of *Lantana camara* L. Leaf Extracts and Soils Invaded by It on the Growth Performance of *Lepidium sativum* L.

**DOI:** 10.1155/2023/6663686

**Published:** 2023-05-13

**Authors:** Likyelesh Gebreyohannes, Meseret C. Egigu, M. Manikandan, J. M. Sasikumar

**Affiliations:** School of Biological Sciences and Biotechnology, College of Natural and Computational Sciences, Haramaya University, Haramaya, P.O. Box 138, Ethiopia

## Abstract

*Lantana camara* is a noxious invasive plant that invades agricultural and natural ecosystems. In the current study, phytotoxicity of hexane and ethanolic leaf extracts of *L. camara* in different concentrations and soils invaded by it on *Lepidium sativum* were investigated under laboratory conditions. Soil toxicity was evaluated by comparing the growth of *L. sativum* on soils sampled from *Lantana*-invaded and *Lantana*-free sites. Results showed that extract concentrations and solvent type and their interaction significantly reduced percent seed germination and seedling growth. Compared to control (distilled water), both hexane and ethanol extracts at 5% w/v concentration significantly reduced percent germination and early seedling growth and completely inhibited seed germination at 10% w/v of hexane leaf extract, suggesting that hexane extract has a greater inhibitory effect than ethanolic extract in all the parameters measured. However, growth performance and seed yield of *L. sativum* grown on soil invaded by *Lantana* did not significantly vary from those grown on soils sampled from noninvaded sites. The results of this study generally showed that though *Lantana* leaf extracts have a direct negative allelopathic effect on *L. sativum*, soils invaded by *Lantana* have no toxic materials in the soil to directly or indirectly inhibit the growth of *L. sativum*. Further field studies on allelopathic effects of *Lantana* on *L. sativum* are recommended.

## 1. Introduction

Allelopathy, an interesting phenomenon, encompasses retardation or stimulation of growth of one plant by the dominance of the other. Allelopathic potential of certain plants causes a detrimental impact on germination and growth parameters of crops and weeds by allelochemicals released by them [1, 2]. Allelochemicals that are released into the environment through leaching, litter decomposition, root exudation, or direct volatilization may inhibit seed germination, seedling growth, and establishment and yield of neighboring crops [3]. They may exert negative impacts through modification of resource consumption capacity, alteration of cell membrane permeability and enzymatic activity, triggering genetic defects, and disturbing photosynthesis of the recipient plants [4].

*Lantana camara*L. (Verbenaceae) is among the top ten invasive plant species [5] growing in many countries under a wide range of climatic conditions and soil types [6–8]. Because of its invasive aptitude, it infests both agricultural and natural ecosystems and is known to be noxious to many associated plant species including crops by releasing allelochemicals [9]. Different parts of *L. camara* (hereafter called *Lantana*) contain allelochemicals, mainly alkaloid and phenolic compounds, which can interfere with seed germination and early seed ling growth of many plant species [2, 5]. The water-soluble allelochemicals of *Lantana* were reported to inhibit early seedling growth of *Oryza sativa*, *Triticum aestivum*, *Vigna sinensis*, *V. mungo*, *V. radiata*, *Cucurbita pepo*, *Abelmoschus esculentus*, *Amaranthus tricolor*, and some weeds [5, 7, 10–13]. Multiple physiological effects including impaired water and nutrient uptake and decreased shoot turgor pressure caused by some phenolic compounds of *Lantana* were reported by Barkosky and Einhellig [14].

*Lantana* is an exotic species in Ethiopia that has wide ecological tolerance to successfully grow in various soil types [7]. It exists in abundance in the eastern part of Ethiopia particularly in Dire Dawa, Hararghe, and Somali regions in the form of hedges around farm lands and along road sides. In addition, it occupies large expanses of unmanaged land by dominating other native range land plant species. With increasing density of *Lantana* in forest and grasslands, declining of species richness is a common phenomenon [15]. Direct negative allelopathic effects of *Lantana* on some crops and its effects on soil quality have been reported previously [9, 16]. However, these effects may vary with plant species and region [16]. Farmers in the Hararghe region of eastern Ethiopia cultivate different vegetables mainly Brassicaceous species including *Lepidium sativum* L. (Brassicaceae). In Ethiopia, *L. sativum* is known as “*fetto*” and is cultivated for its medicinal and food values [17]. However, information related to the allelopathic effects of *Lantana* on the growth of *L. sativum* is lacking. We hypothesized that *Lantana* leaf extracts and soils invaded by it have negative allelopathic effects on *L. sativum*. Therefore, this research was initiated with the objective of evaluating the allelopathic effect of *Lantana* leaf extract and residual toxicity of soils sampled from land invaded by *Lantana* on *L. sativum*. *Lepidium sativum* was selected as a model plant for its short life cycle and sensitivity to allelochemicals besides its great importance as a food and medicinal plant in Ethiopia.

## 2. Materials and Methods

### 2.1. Plant Material and Soil Sampling Site

In the Hararghe region, eastern part of Ethiopia, *L. camara* is a widely distributed weed that is found mostly around farm lands as hedges and along road sides. It is also seen encroaching on unmanaged areas covered by natural vegetation. Thus, fresh leaves and soil samples were collected from around Haramaya University (9°24′53.13″N and 42°01′55.69″E), East Hararghe zone of Oromia regional state, Ethiopia. The sampling field was a mosaic land covered by thickets of *Lantana* interspersed with patches of small shrubs, grasses, and forbs and subjected to open grazing by livestock. Crops such as sorghum, maize, and khat (*Catha edulis*) and vegetables, mainly Brassicaceous spp., are also cultivated by local farmers adjacently.

### 2.2. Allelopathic Plant

In the Petri plate testing, *L. camara* served as an allelopathic plant and solvent extracts of the leaves were used for testing its inhibitory potential.

### 2.3. Target Plant

*L. sativum* was the target plant and its seeds were collected from the fields of Bate village, Haramaya district.

### 2.4. Design for *Lantana* Leaf and Soil Sampling

The sampling design used by Fan et al. [9] was followed in this study. Four sampling points that were densely covered by stands of *Lantana* were purposively marked to take leaf and soil samples. The four sampling points (replicates) were far apart from each other by at least 25 m and had roughly similar slopes and topographies with sites adjacent to them covered by patches of grasses, forbs, and other shrubs but not *Lantana*. From each replicate sampling point, fresh leaves of focal *Lantana* plants were collected and placed in the same plastic bag. Soil samples were also taken from the four sampling points starting from under *Lantana* canopy (UC) and in a distance gradient within 1-2 and 2-3 m limits outside the canopy influence in the same direction. Soil samples were taken from the upper 20 cm using an auger in a 1 m × 1 m quadrat from each distance gradient. In each quadrat, a soil sample was taken from the four corners and center of the quadrat and pooled to form a composite soil. The sampled soils were used for the cultivation of *L. sativum* in pots in a greenhouse.

### 2.5. *Lantana* Leaf Extraction

*Lantana* leaves sampled from focal plants at for sampling points were first thoroughly washed with distilled water and air dried in the laboratory at room temperature for about ten days. Air-dried leaves were powdered together using a mortar and pestle. The powder (100 g) was mixed with 300 ml of organic solvents (ethanol and hexane separately) and left on the table in the laboratory for 24 hours with intermittent stirring using a glass rod [18]. After 24 hours of soaking, the extract was filtered under suction using Whatman No. 1 filter paper and the solvent in the filtrate was evaporated under reduced pressure using a rotary evaporator at 55°C to remove the solvent, and the dried extract was stored in a refrigerator until bioassay.

### 2.6. Extracts' Allelopathic Bioassay

In order to test the impact of *Lantana*'s leaf extracts on seed germination parameters of *L. sativum*, the dried extracts (5 and 10 g) were reconstituted by dissolving in 100 mL of distilled water to have 5 and 10% (w/v) concentrations. Prior to the germination bioassay, seeds of *L. sativum* were surface sterilized using 15% sodium hypochlorite for 20 min and rinsed in distilled water. Thereafter, 10 seeds were placed on Whatman No. 1 filter paper that received 15 mL of 5 and 10% hexane and ethanolic extracts (separately) in Petri dishes of 9 cm diameter. Distilled water of equal volume to the extract was used as negative control. The seeds were randomly assigned to different treatment levels, and Petri dishes were arranged on a table in a laboratory in 5 replicates and incubated for days to complete germination. Petri dishes were made to get more or less the same amount and types of light throughout the incubation period and moved around to avoid the position effect. Moisture in the Petri dishes was maintained by adding 2 mL of the extract or distilled water every 2 days. After 10 days of incubation, percent germination and shoot and root lengths of *L. sativum* were measured.

### 2.7. Impact of Soils Invaded by *Lantana*

Soil samples of each distance gradient, i.e., UC, 1-2 m and 2-3 m distances outside the influence of *Lantana* canopy obtained from the four sampling points (replicates) were filled in separate plastic pots (4 pots per treatment) having a surface area of 380 cm^2^. After sowing 10 seeds of the test plant in each pot, the pots were arranged on a table in the greenhouse. The plants were watered with tap water regularly to keep soils always moist, but were not fertilized. The plants were maintained until maturity (4 months and 2 weeks), and parameters such as germination percentage, aboveground plant height measured at harvest, shoot dry weight at harvest, seed yield at harvest, and thousand seeds weight were measured eventually.

### 2.8. Analyses of Selected Soil Chemical Parameters

Soil composites sampled from different sites as indicated above were analyzed for soil organic matter (SOM), total nitrogen (TN), pH, and available phosphorus following standard methods. Total soil nitrogen was determined by following the method indicated by [19] using the micro-Kjeldahl distillation and titration method. The SOM was analyzed using the Walkley–Black procedure [20]. Soil pH was determined with a pH meter (pH rex-2 lei-ci, Shanghai) with 1 : 2.5 w/v (soil: distilled water) ratio. The available *P* was determined calorimetrically using the molybdenum-blue method [21].

### 2.9. Statistical Analysis

A general linear model was employed to analyze the impacts of the main effects and their interaction on seed germination and seedling growth. Effects of soils on growth parameters were analyzed using one-way ANOVA. Statistical software SPSS for Windows 16.0 (SPSS, Chicago, IL, USA) was used to analyze all the data. Differences between means were separated using the LSD test, and *P* value less than 0.05 was considered as statistically significant.

## 3. Results

### 3.1. Effects of *Lantana* Leaf Extracts on Germination Percentage of *L. sativum* Seeds

**Figure 1:**
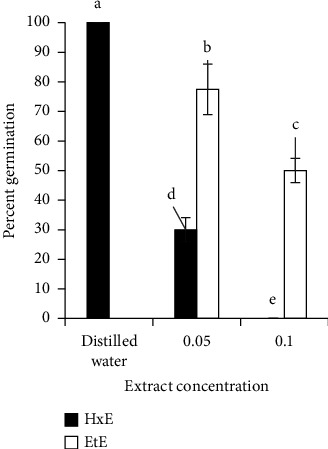
Germination percentage of seeds of *L. sativum* treated with different concentrations of leaf hexane (HxE) and ethanol extracts (EtE) of *Lantana*. Values are mean ± S.E, *n* = 5. *Note*. Black solid bar graph for 10% w/v hexane extract did not appear as value is zero.

Extract concentrations and solvents used to make extracts were the main effects. The extracts, at all levels of concentration, significantly (df = 2, *F* = 48.257, and *P* < 0.001) inhibited *L. sativum*'s seed germination when compared with control and the germination inhibitory effect increased with increasing extract concentration (Figure 1). Seed germination was also significantly (df = 1, *F* = 26.737, and *P* < 0.001) varied with the type of solvent used to make the extraction with hexane extract exhibiting the highest inhibitory effect than ethanolic extract (Figure 1). That is, hexane extract was superior to ethanolic extract in inhibiting percent seed germination with complete inhibition of seed germination at 10% (w/v) concentration.

### 3.2. Effects on Shoot and Root Lengths of *L. sativum*

**Figure 2:**
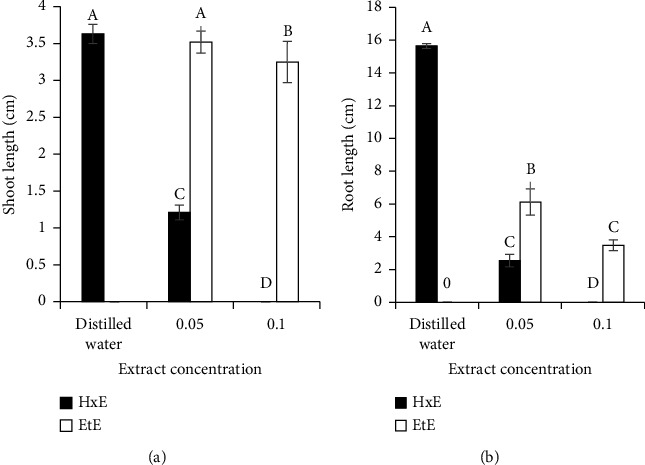
Shoot (a) and root (b) lengths of *L. sativum* treated with different concentrations of leaf hexane (HxE) and ethanol extracts (EtE) of *Lantana.* Values are mean ± S.E, *n* = 5. *Note*. Black solid bar graph for 10% w/v hexane extract did not appear as value is zero.

Shoot and root growths of *L. sativum* were significantly (*P* < 0.05) inhibited by extract concentration, extraction solvent, and their interaction (Figures 2(a) and 2(b)). Shoot and root growth inhibitions were more pronounced with increasing extract concentration. Hexane-extracted solution had significantly (*P* < 0.05) higher negative impact than ethanol-extracted solution (Figures 2(a) and 2(b)).

### 3.3. Impact of Soils from *Lantana*-Invaded Site on Growth *of L. sativum*

No significant difference was observed between *L. sativum* grown on soils invaded by *Lantana* and those grown on *Lantana*-free soils in percent germination and all agronomic parameters including the aboveground plant height (PH), shoot dry weight (SDW), seed yield (SY), and 1000 seeds weight (TSW) (Table 1).

### 3.4. Selected Chemical Properties of Soils Sampled from Different Sites

Results of the analyses of the selected soil chemical features showed that there were no significant (*P* > 0.05) differences in SOM, pH, AP, and TN between the different sampling points along the distance gradient from the influence of *Lantana* canopy (Table 2).

## 4. Discussion

### 4.1. Leaf Extracts of *Lantana* Are Toxic to *L. sativum*

Our most significant observations were that the direct application of leaf extracts of *Lantana* had negative effects on *L. sativum*'s seed germination and early seedling growth. Inhibitory effects of allelochemicals at the germination and early seedling growth stage may predispose crops to other abiotic and biotic stresses and hence reduce their productivity [22]. In this study, percent germination and seedling growth were inhibited more by the highest extract concentration. This suggests that *Lantana* leaf possesses secondary compounds that have a negative allelopathic effect on the tested plant. The direct allelopathic effect of *Lantana* was reported previously by different researchers [10, 12, 23–27] on several other crop species. The phytotoxicity of leaf extract may be attributed to secondary compounds, mainly to complex interaction of some alkaloids and phenolic compounds [28]. In addition to phenolics, a report by Kong et al. [29] indicates that triterpenes, lantadene A and B (pentacyclic triterpenoids), isolated from *Lantana* are also potent allelochemicals. The triterpenoids had inhibitory effects on germination, radical growth, and function of photosystem II [30]. Both hexane and ethanolic leaf extracts significantly reduced percent seed germination and early seedling growth at 5% (w/v) concentration compared to the control. However, hexane leaf extract had a more inhibitory effect than ethanolic leaf extract as it completely inhibited percent seed germination at 10% (w/v). This shows that solvents of different polarities have different potential to extract compounds of different profiles showing different activities [23]. The outcomes of the present study also showed that the inhibitory effects of *Lantana* leaf extracts on the germination of *L. sativum*'s seed was concentration-dependent and the higher concentration possessed pronounced inhibitory effects than the lower concentration. The matching tendency was observed in some previous investigations [31–33].

Seed germination may be inhibited due to hampered resource mobilization by allelochemicals during the early stages of seed germination [34]. It is also possible that allelochemicals such as some phenolic compounds impair the synthesis and/or activity of gibberellic acid [35], which regulates the production of amylase [36], so that seed germination is negatively affected. Einhellig [35] reported that allelochemicals decrease elongation, expansion, and division of cells, which are growth prerequisites. The inhibition of shoot and root lengths by *Lantana* extracts also suggests the occurrence of some allelochemicals that interfere with important metabolic activities capable of promoting cell division and elongation. Sasikumar et al. [37] reported the inhibitory effects of phenolic compounds on the phosphorylation pathway, Mg activation, and ATPase activity. They also mentioned that allelochemicals might decrease the synthesis of total carbohydrates, proteins, and nucleic acids (DNA and RNA), hence negatively affecting growth. Compared to the control, root length was six-fold reduced, whereas shoot growth was reduced by three-fold only at 5% w/v of leaf hexane extract. This may be attributed to more impairment of endogenous plant growth hormones synthesis and/or activity in the root by allelochemicals.

### 4.2. Impact of Soils from *Lantana-*Invaded Site on Growth of *L. sativum*

Allelochemicals may enter into soils invaded by the donor plant through different means including tissue decomposition, aerial leachates from a donor plant, and exudation from roots. We expected that these chemicals will also have an inhibitory effect after entering into the soil. However, *Lantana*-invaded soils had no significantly different effects on growth parameters measured as compared to soils used from *Lantana*-free sites. The fact that soils invaded by *Lantana* had no inhibitory effect on the test plant suggests less accumulation of allelochemicals in the soils. It is also possible that allelochemicals might have been degraded or transformed into some other nontoxic forms [38–40] in soils by microbial and physicochemical processes. Achhireddy and Singh [41] and Sahid and Sugau [2] reported that soils invaded by *Lantana* have no inhibitory effect when used to grow other crops as opposed to direct extract application to the seeds. Apart from direct toxicity to recipient plants, allelochemicals may have a negative influence on the amount and availability of plant essential nutrients in the soil, hence reducing the plant' growth. We also analyzed selected soil chemical parameters to check if there is a difference between sites along the distance gradient from *Lantana*-invaded to *Lantana*-free sites. From the results, the invasion of a site by *Lantana* did not result in a significant difference in soil chemical properties. Here, it is important to note that we analyzed only a few selected chemical parameters (few macronutrients and pH), which requires further investigation of many other macro and micronutrients. In support of our findings, previous studies by Fan et al. [9] and Osunkoya and Perrett [16] showed that the invasion of soil by *Lantana* has no negative effect at least on some major soil macronutrients such as phosphorus, nitrogen, and organic carbon.

## 5. Conclusions

In conclusion, this study showed that *Lantana* leaf extracts hamper germination and early seedling growth of *L. sativum*, but the soil invaded by *Lantana* has no negative impact on *L. sativum* growth and seed yield. Moreover, our results show that soil invasion by *Lantana* is less likely to have a negative impact on soil nutrient status to reduce growth performance of the tested plant.

## Figures and Tables

**Table 1:** Impact of soils sampled from under *L. camara* canopy and *Lantana*-free sites on growth performance and grain yield of *L. sativum.*

Germination parameters	Soil sampling position			*F* value	*P* value
	UC	1-2 m	2-3 m		
Germination (%)	95.50 ± 1.55^a^	93.00 ± 0.90^a^	93.00 ± 0.90^a^	0.613	0.62
PH (cm)	47.66 ± 0.96^a^	45.54 ± 1.80^a^	37.61 ± 0.85^a^	27.412	0.07
SDW (gm)	3.91 ± 0.05^a^	2.91 ± 0.10^a^	2.32 ± 0.09^a^	1.538	0.278
SY (kg·ha^−1^)	3633.33 ± 120.18^a^	3680.00 ± 75.73^a^	3616.67 ± 2.5^a^	0.146	0.867
TSW	2.07 ± 0.03^a^	2.10 ± 0.06^a^	1.83 ± 0.03^b^	10.83	0.05

*Note*. Values are mean ± SE, *n* = 4. UC, soil sampling position was from under the canopy of *Lantana*; 1-2 m, soil sampling position was within a distance of >1 m and <2 m outside the influence of *Lantana* canopy; 2-3 m, soil sampling position was within a distance of >2 m and <3 m outside the influence of *Lantana* canopy; PH, aboveground plant height measured at harvest; SDW, shoot dry weight at harvest; SY, seed yield at harvest; TSW, thousand seeds weight. Values followed by similar lower case letters in a row are not significantly different, whereas those represented with different letters are significantly different at *P* < 0.05.

**Table 2:** Chemical properties of the soil samples taken at distant gradients from the influence of *Lantana* canopy.

Parameters	UC	1-2 m	2-3 m
pH	7.02 ± 0.10^a^	7.20 ± 0.12^a^	7.21 ± 0.03^a^
SOM (%)	1.18 ± 0.27^b^	1.23 ± 0.10^a^	1.29 ± 0.13^a^
Available *P* (ppm)	14.45 ± 6.68^a^	14.62 ± 2.25^a^	16.62 ± 7.29^a^
Total *N* (%)	0.08 ± 0.02^a^	0.10 ± 0.03^a^	0.09 ± 0.02^a^

*Note.* Values are mean ± SE, *n* = 4. UC, soil sampling position was from under the canopy of *Lantana*; 1-2 m, soil sampling position was within a distance of >1 m and <2 m outside the influence of *Lantana* canopy; 2-3 m, soil sampling position was within a distance of >2 m and <3 m outside the influence of *Lantana* canopy. Values followed by similar lower case letters in a row were not significantly different at *P* < 0.05.

## Data Availability

All relevant data are included within the article.
